# Impact of comorbidities on the serological response to COVID-19 vaccination in a Taiwanese cohort

**DOI:** 10.1186/s12985-023-02056-5

**Published:** 2023-06-02

**Authors:** Chung-Feng Huang, Tyng-Yuan Jang, Ping-Hsun Wu, Mei-Chuan Kuo, Ming-Lun Yeh, Chih-Wen Wang, Po-Cheng Liang, Yu-Ju Wei, Po-Yao Hsu, Ching-I Huang, Ming-Yen Hsieh, Yi-Hung Lin, Hui-Hua Hsiao, Chin-Mu Hsu, Chien-Tzu Huang, Chun-Yuan Lee, Yen-Hsu Chen, Tun-Chieh Chen, Kun-Der Lin, Shuo-Hung Wang, Sheng-Fan Wang, Jee-Fu Huang, Chia-Yen Dai, Wan-Long Chuang, Ming-Lung Yu

**Affiliations:** 1 Hepatobiliary Division, Department of Internal Medicine, Kaohsiung Medical University Hospital, Kaohsiung Medical University, 100 Tzyou Road, Kaohsiung City, 807 Taiwan; 2 Ph.D. Program in Translational Medicine, College of Medicine, Kaohsiung Medical University, Kaohsiung, Taiwan; 3 Academia Sinica, Taipei, Taiwan; 4 Faculty of Medicine, Kaohsiung Medical University, Kaohsiung, Taiwan; 5 Division of Nephrology, Department of Internal Medicine, Kaohsiung Medical University Hospital, Kaohsiung Medical University, Kaohsiung, Taiwan; 6 Department of Internal Medicine, Kaohsiung Municipal Siaogang Hospital, Kaohsiung Medical University, Kaohsiung, Taiwan; 7 Department of Internal Medicine, Kaohsiung Municipal Ta-Tung Hospital, Kaohsiung Medical University, Kaohsiung, Taiwan; 8 Division of Hematology and Oncology, Department of Internal Medicine, Kaohsiung Medical University Hospital, Kaohsiung Medical University, Kaohsiung, Taiwan; 9 Division of Infectious Diseases, Department of Internal Medicine, Kaohsiung Medical University Hospital, Kaohsiung Medical University, Kaohsiung, Taiwan; 10 School of Medicine, M.Sc. Program in Tropical Medicine, Graduate Institute of Medicine, College of Medicine, Kaohsiung Medical University, Kaohsiung, Taiwan; 11 School of Medicine, Graduate Institute of Medicine, Sepsis Research Center, Center of Tropical Medicine and Infectious Diseases, Kaohsiung Medical University, Kaohsiung, Taiwan; 12 Department of Biological Science and Technology, College of Biological Science and Technology, National Chiao Tung University, Hsinchu, Taiwan; 13 School of Medicine, College of Medicine, National Sun Yat-Sen University, Kaohsiung, Taiwan; 14 The Lin’s Clinic, Kaohsiung, Taiwan; 15 Department of Medical Laboratory Science and Biotechnology, Kaohsiung Medical University, Kaohsiung, Taiwan; 16 School of Medicine, and Doctoral Program of Clinical and Experimental Medicine, College of Medicine, Center of Excellence for Metabolic Associated Fatty Liver Disease, National Sun Yat-Sen University, Kaohsiung, Taiwan; 17 Division of Hepato-Gastroenterology, Department of Internal Medicine, Kaohsiung Chang Gung Memorial Hospital, Kaohsiung, Taiwan

**Keywords:** COVID-19, Comorbidity, Vaccine, Response, Taiwan

## Abstract

**Background/Aims:**

Vaccination against severe acute respiratory syndrome coronavirus 2 (SARS-CoV-2) is one of the best policies to control COVID-19 pandemic. The serological response to COVID-19 vaccination in Taiwanese patients with different comorbidities is elusive.

**Methods:**

Uninfected subjects who received 3 doses of mRNA vaccines (BNT162b2 [Pfizer-BioNTech, BNT] and mRNA-1273 [Moderna]), viral vector-based vaccines (ChAdOx1-S (AZD1222, AZ) or protein subunit vaccines (Medigen COVID-19 vaccine) were prospectively enrolled. The SARS-CoV-2-IgG spike antibody level was determined within three months after the 3rd dose of vaccination. The Charlson Comorbidity Index (CCI) was applied to determine the association between vaccine titers and underlying comorbidities.

**Results:**

A total of 824 subjects were enrolled in the current study. The proportions of CCI scores of 0–1, 2–3 and > 4 were 52.8% (n = 435), 31.3% (n = 258) and 15.9% (n = 131), respectively. The most commonly used vaccination combination was AZ–AZ–Moderna (39.2%), followed by Moderna–Moderna–Moderna (27.8%). The mean vaccination titer was 3.11 log BAU/mL after a median of 48 days after the 3rd dose. Factors associated with potentially effective neutralization capacity (IgG level ≥ 4160 AU/mL) included age ≥ 60 years (odds ratio [OR]/95% confidence interval [CI]: 0.50/0.34–0.72, *P* < 0.001), female sex (OR/CI: 1.85/1.30–2.63, *P* = 0.001), Moderna–Moderna-based vaccination (compared to AZ–AZ-based vaccination, OR/CI: 6.49/3.90–10.83, *P* < 0.001), BNT–BNT-based vaccination (compared to AZ–AZ-based vaccination, OR/CI: 7.91/1.82–34.3, *P* = 0.006) and a CCI score ≥ 4 (OR/CI: 0.53/0.34–0.82, *P* = 0.004). There was a decreasing trend in antibody titers with increasing CCI scores (trend *P* < 0.001). Linear regression analysis revealed that higher CCI scores (β: − 0.083; 95% CI: − 0.094–0.011, *P* = 0.014) independently correlated with low IgG spike antibody levels.

**Conclusions:**

Subjects with more comorbidities had a poor serological response to 3 doses of COVID-19 vaccination.

## Introduction

Severe acute respiratory syndrome coronavirus 2 (SARS-CoV-2), which was first identified in China in 2019, has caused the coronavirus disease 2019 (COVID-19) pandemic [1, 2]. COVID-19 has now spread worldwide, affecting over 600 million people as of September 2022 and claiming the lives of nearly 6 million people worldwide. The dominant viral strains were the Alpha and Beta variants before 2021 [3], and the Delta variant cluster emerged in June 2021 [4]. Due to the strict preventive strategies, the implementation of meticulous border control measures and advanced deployment adopted in Taiwan, there were very few transient outbreaks in 2020 and 2021 [5–7]. With the emergence of the Omicron variant and the wide adoption of COVID-19 vaccination [8], Taiwan has transitioned to an era of viral coexistence since May 2022.

Apart from relying on social distancing, hygiene measures and antiviral allocations, the most effective way to protect against severe COVID-19 is by developing vaccines. The introduction of an effective vaccine against SARS-CoV-2 has been a worldwide effort, and as of 2022, there are dozens of vaccines in clinical trials with over 200 in various stages of development. A candidate vaccine against SARS-CoV-2 might act against acquisition or transmission and reduce hospitalization and mortality in vulnerable populations. The use of serological markers such as spike antibodies or neutralizing antibodies against COVID-19 may provide alternative evidence of the efficacy of a vaccine [9]. Currently, there are four approved COVID-19 vaccines in Taiwan, including two mRNA vaccines (BNT162b2 [Pfizer-BioNTech, BNT] and mRNA-1273 [Moderna]), a nonreplicating viral vector vaccine (ChAdOx1-S (AZD1222, AZ) and a protein subunit vaccine (Medigen COVID-19 vaccine, MVC). The vaccination response may vary among subjects and may be suboptimal in relatively immunocompromised subjects [10–13]. Until now, there have been no real-world data presented regarding the effectiveness of COVID-19 vaccines in Taiwanese people. Imperatively, whether there are differences in vaccine responses among subjects who possess different comorbidities remains elusive. Herein, we aimed to address this issue by prospectively enrolling Taiwanese subjects with well-characterized underlying or concurrent diseases after three doses of COVID-19 vaccination.

## Methods

### Patients

Participants who received 3 doses of COVID-19 vaccination were prospectively and consecutively recruited in a medical center in Taiwan from 2021 to 2022. Subjects were excluded if they met the following criteria: (1) were aged less than 20 years; (2) were inoculated with less than three doses of COVID-19 vaccination; (3) had a history of COVID-19 infection; (4) had a contact history of COVID-19 infection; and (5) had a foreign travel history in the past 1 year. Participants were invited and recruited to join the study during outpatient department visits at Kaohsiung Medical University Hospital. Information on travel history and contact history was collected during a case interview by a well-constructed questionnaire. Histories of recent foreign travel and COVID-19 infection were altered simultaneously in the electrical medical record by the Bureau of National Health Insurance, Taiwan and were displayed automatically on the screen while at the clinics. In 2022, Taiwan reported the local clustering of 83 cases for the first time on March 27th, and then encountered the COVID-19 pandemic in the community since May 2022. Subjects who completed the 3rd dose of vaccination after March 2022 were also excluded to avoid recruiting previously infected subjects. Since there is no inactivated COVID-19 vaccine being launched in Taiwan, blood samples were further tested for nucleocapsid antibody to identify those who might have been infected. All patients provided written informed consent. The ethics committee of the Kaohsiung Medical University Hospital approved the study.

### Laboratory analyses and comorbidity interpretation

The SARS-CoV-2-IgG antibodies to spike protein and nucleocapsid (cutoff > = 1.4 S/C) were measured by chemiluminescent microparticle immunoassay (CMIA) (Abbott, ARCHITECT SARS-CoV-2 IgG II). The anti-spike protein antibody is highly correlated with the WHO International Standard (binding antibody unit, BAU) (Abbott: BAU/mL = 0.142 × AU/mL). Blood samples and antibody titers were checked 1–3 months after the 3rd COVID-19 vaccination. The current or past medical history was obtained from the predesigned questionnaire as well as the electrical medical records during blood sampling. The Charlson Comorbidity Index (CCI) was applied to address the association of comorbidity with vaccine response [14].

### Statistical analyses

Frequencies were compared between groups using the χ^2^ test with Yates correction or Fisher’s exact tests. Variable means are presented as the means ± standard deviations and were compared using analysis of variance, Student’s t test, or the nonparametric Mann–Whitney test. The potentially effective neutralization capacity was defined as having an IgG level ≥ 4160 AU/mL [15]. Stepwise logistic regression analysis was applied to assess the factors associated with high IgG levels by analyzing the covariants with *P* values < 0.1 in the univariate analysis. Linear regression analysis was used to assess the factors correlated with the levels of IgG spike antibodies. Statistical analyses were performed using the SPSS 20 statistical package (SPSS, Chicago, IL, USA). All statistical analyses were based on two-sided hypothesis tests, with a statistical significance of *P* < 0.05.

## Results

### Patient characteristics

A total of 824 subjects were enrolled in the current study. The mean age was 58.9 years (range: 22–91 years); men comprised 48.7% (n = 401) of the cohort. The proportions of CCI scores of 0–1, 2–3 and > 4 were 52.8% (n = 435), 31.3% (n = 258) and 15.9% (n = 131), respectively. The most commonly used vaccination combination was AZ–AZ–Moderna (39.2%), followed by Moderna–Moderna–Moderna (27.8%) and AZ–AZ–BNT (14.7%). The median IgG level was 9812 AU/mL (range 87–236,599 AU/mL), which corresponded to a titer of 3.11 log BAU/mL with a median of 48 days after the 3rd dose vaccination (Table 1 and Table 2). Of the 780 (94.6%) patients with available samples for testing nucleocapsid antibody, only one (0.12%) patient was tested positive with the titer of 1.6 S/C.

**Table 1:** Characteristics of the subjects

	All patients (n = 824)
Age (years, mean (SD))	58.9 (13.7)
Male, n (%)	401 (48.7)
BMI (kg/m^2^, mean (SD))	24.3 (3.0)
Medical history	
Dyslipidemia, n/N (%)	128/822 (15.6)
Hypertension, n/N (%)	278/822 (33.8)
Diabetes, n/N (%)	147/822 (17.9)
Chronic kidney disease, n/N (%)	166/822 (20.2)
HIV Infection, n/N (%)	5/822 (0.6)
Chronic liver disease, n/N (%)	552/822 (67.2)
Cardiovascular disease, n/N (%)	17/818 (2.1)
Cerebrovascular disease, n/N (%)	10/818 (1.2)
COPD, n/N (%)	14/818 (1.7)
History of malignancy, n/N (%)	101/822 (12.3)
Charlson comorbidity index (mean (SD))	2.0 (1.8)
Charlson Comorbidity index (median (range))	1 (0–13)
Charlson comorbidity index	5.44 (0.98)
0	130 (15.8)
1	305 (37.0)
2	146 (17.7)
3	112 (13.6)
4	46 (5.6)
5	36 (4.4)
≧ 6	49 (5.9)
SARS-CoV-2 IgG (AU/mL, median (range))	9812 (87–236,599)
SARS-CoV-2 IgG (log BAU/mL, mean (SD))	3.11 (0.46)
Time of blood testing after the 3rd vaccination (days, mean (SD))	49.1 (20.4)

*SD*, standard deviation; *BMI*, body mass index; *HIV*, Human immunodeficiency virus; *COPD*, Chronic pulmonary obstructive disease

**Table 2:** SARS-CoV-2 IgG titers between/among groups with different characteristics

	SARS-CoV-2 IgG titers (log BAU/mL, mean (SD))	*P* value
Age, years		0.49
< 60 (n = 390)	3.12 (0.40)	
≧ 60 (n = 434)	3.10 (0.51)	
Gender		0.02
Male (n = 401)	3.07 (0.48)	
Female (n = 423)	3.15 (0.45)	
BMI		0.03
< 24 kg/m^2^ (n = 387)	3.07 (0.48)	
≧ 24 kg/m^2^ (n = 409)	3.14 (0.45)	
Dyslipidemia		0.36
Yes (n = 128)	3.07 (0.52)	
No (n = 694)	3.11 (0.45)	
Hypertension		0.71
Yes (n = 278)	3.10 (0.45)	
No (n = 544)	3.12 (0.49)	
Diabetes		0.64
Yes (n = 147)	3.09 (0.53)	
No (n = 675)	3.11 (0.45)	
Chronic kidney disease		< 0.001
Yes (n = 166)	2.97 (0.53)	
No (n = 656)	3.14 (0.44)	
Chronic liver disease*		0.12
Yes (n = 552)	3.13 (0.45)	
No (n = 270)	3.07 (0.49)	
History of malignancy		0.25
Yes (n = 101)	3.04 (0.58)	
No (n = 721)	3.11 (0.45)	
Charlson comorbidity index		< 0.001 (CCI 0–1 vs. 2–3 vs. ≧ 4)
0 (n = 130)	3.14 (0.38)	
1 (n = 305)	3.14 (0.40)	
2 (n = 146)		3.09 (0.48)
3 (n = 112)	3.12 (0.50)	
≧ 4 (n = 131)	3.00 (0.56)	

*SD*, standard deviation; *BMI*, body mass index; *CCI*, Charlson Comorbidity Index

*liver cirrhosis (n = 61) versus non-liver cirrhosis (n = 736): 3.14 + 0.59 log BAU/mL versus 3.11 + 0.45 log BAU/mL (*P* = 0.59)

### SARS-CoV-2 IgG titers in subjects with different subgroups and vaccines

The anti-spike IgG levels of AZ–AZ-based, Moderna–Moderna-based, MVC–MVC-based and BNT–BNT-based vaccines were 2.98 log BAU/mL, 3.34 log BAU/mL, 3.01 log BAU/mL and 3.37 log BAU/mL, respectively (Table 2). The anti-spike IgG titer was significantly higher in females (3.15 vs. 3.07 log BAU/mL, *P* = 0.02), subjects with a BMI > 24 kg/m^2^ (3.14 vs. 3.07 log BAU/mL, *P* = 0.03), and patients without chronic kidney disease (3.14 vs. 2.97 log BAU/mL, *P* < 0.001). There was a decreasing trend in the antibody titer with increasing CCI scores (*P*_trend_ < 0.001) (Table 2). Compared to subjects with AZ–AZ-based vaccination (2.98 log BAU/mL), a higher anti-spike IgG level was noted in subjects with Moderna–Moderna-based vaccination (3.34 log BAU/mL) and BNT–BNT-based vaccination (3.37 log BAU/mL) (Table 3).

**Table 3:** SARS-CoV-2 IgG titers between/among groups with different vaccination

1st dose-2nd dose-3rd dose	N (%)	SARS-CoV-2 IgG titer (log BAU/mL, mean (SD))
AZ-based	475 (57.6)	2.98 (0.46)^a^
AZ–AZ–AZ	1 (0.1)	3.63
AZ–AZ–Moderna	323 (39.2)	3.03 (0.45)
AZ–AZ–Medigen	30 (3.6)	2.57 (0.34)
AZ–AZ–BNT	121 (14.7)	2.94 (0.44)
Moderna-based	253 (30.7)	3.34 (0.39)^b^
Moderna–Moderna–AZ	1 (0.1)	3.71
Moderna–Moderna–Moderna	229 (27.8)	3.38 (0.38)
Moderna–Moderna–Medigen	11 (1.3)	2.84 (0.31)
Moderna–Moderna–BNT	12 (1.5)	3.05 (0.37)
Medigen-based	20 (2.4)	3.01 (0.45)^c^
Medigen–Medigen–Moderna	4 (0.5)	3.44 (0.41)
Medigen–Medigen–Medigen	10 (1.2)	2.80 (0.34)
Medigen–Medigen–BNT	6 (0.7)	3.06 (0.45)
BNT-based	33 (4.0)	3.37 (0.33)^d^
BNT–BNT–Moderna	15 (1.8)	3.50 (0.29)
BNT–BNT–Medigen	1 (0.1)	3.65
BNT–BNT–BNT	17 (2.1)	3.24 (0.32)
Other vaccination combination	43 (5.2)	3.00 (0.45)

*SD*, standard deviation; *BNT*, BNT162b2 [Pfizer-BioNTech]; *Moderna*, mRNA-1273 [Moderna]; *AZ*, ChAdOx1-S (AZD1222); *Medigen*, Medigen COVID-19 vaccine

a versus b: *P* < 0.001. a versus c. *P* = 0.79. a versus d: *P* < 0.001

### Factors associated with high SARS-CoV-2 IgG titers

Six hundred thirty-six (77.2%) of the 824 subjects had IgG titers equal to or greater than 4160.

AU/mL, which may be considered as having neutralization capacity. Compared to the group whose titers were less than 4160 AU/mL, the group with high antibody titers had a smaller proportion of older subjects (age ≥ 60 years; 50.5% vs. 60.1%, *P* = 0.03), subjects with diabetes (15.9% vs. 24.6%, *P* = 0.01), subjects with chronic kidney disease (17.2% vs. 30.5%, *P* < 0.001) and subjects with underlying malignancy (11.0% vs. 16.6%, *P* = 0.04) and a larger proportion of females (54.7% vs. 39.9%, *P* < 0.001) and subjects with CCI scores < 4 (86.6% vs. 75.0%, *P* < 0.001) (Table 4). Subjects who received Moderna–Moderna-based vaccination and BNT–BNT-based vaccination and had a larger proportion of high SARS-CoV-2 IgG titers than those who received AZ–AZ-based vaccination (91.7% and 93.9% vs. 68.6%, *P* < 0.001, respectively). Logistic regression analysis revealed that factors associated with high antibody titers were an age ≥ 60 years (odds ratio [OR]/95% confidence interval [CI]: 0.50/0.34–0.72, *P* < 0.001), female sex (OR/CI: 1.85/1.30–2.63, *P* = 0.001), Moderna–Moderna-based vaccination (compared to AZ–AZ-based vaccination, OR/CI: 6.49/3.90–10.83, *P* < 0.001), BNT–BNT-based vaccination (compared to AZ–AZ-based vaccination, OR/CI: 7.91/1.82–34.3, *P* = 0.006) and a CCI score ≥ 4 (OR/CI: 0.53/0.34–0.82, *P* = 0.004) (Table 4). Linear regression analysis also demonstrated that CCI scores inversely correlated to the titer (β: − 0.083; 95% CI: − 0.094–0.011, *P* = 0.014) (Table 5).

**Table 4:** Factors associated with high SARS-CoV2-IgG spike antibody level

	SARS-CoV-2 IgG titers ≧ 4160 AU/mL (n = 636)	SARS-CoV-2 IgG titers < 4160 AU/mL (n = 188)	*P* value	Logistic regression analysis		
				OR	95% CI	*P* value
Age ≧ 60 years, n (%)	321 (50.5)	113 (60.1)	0.03	0.50	0.34–0.72	< 0.001
Female, n (%)	348 (54.7)	75 (39.9)	< 0.001	1.85	1.30–2.63	0.001
BMI (kg/m^2^, mean (SD))	24.4 (3.9)	24.0 (4.2)	0.36			
Medical history						
Dyslipidemia, n/N (%)	98/635 (15.4)	30/187 (16.0)	0.84			
Hypertension, n/N (%)	211/635 (33.2)	67/187 (35.8)	0.51			
Diabetes, n/N (%)	101/635 (15.9)	46/187 (24.6)	0.01			
Chronic kidney disease, n/N (%)	109/635 (17.2)	57/187 (30.5)	< 0.001			
HIV infection, n/N (%)	3/635 (0.5)	2/187 (1.1)	0.32			
Chronic liver disease, n/N (%)	430/635 (67.7)	122/187 (65.2)	0.53			
Cardiovascular disease, n/N (%)	15/632 (2.4)	2/186 (1.1)	0.39			
Cerebrovascular disease, n/N (%)	6/632 (0.9)	4/186 (2.2)	0.25			
COPD, n/N (%)	10/632 (1.6)	4/186 (2.2)	0.53			
History of malignancy, n/N (%)	70/635 (11.0)	31/187 (16.6)	0.04			
Vaccination type, the first 2 doses						
AZ–AZ	326 (51.3)	149 (79.3)		1		
Medigen–Medigen	13 (2.0)	7 (3.7)		0.82	0.31–2.16	0.69
BNT–BNT	31 (4.9)	2 (1.1)		7.91	1.82–34.3	0.006
Moderna–Moderna	232 (36.5)	21 (11.2)		6.49	3.90–10.83	< 0.001
CCI			< 0.001			
< 4	552 (86.6)	141 (75.0)		1		
≧ 4	84 (13.2)	47 (25.0)		0.53	0.34–0.82	0.004

*SD*, standard deviation; *BMI*, body mass index; *CKD*, chronic kidney disease; *HCC*, hepatocellular carcinoma; *CCI*, Charlson Comorbidity Index; *BNT*, BNT162b2 [Pfizer-BioNTech]; *Moderna*, mRNA-1273 [Moderna]; *AZ*, ChAdOx1-S (AZD1222)

**Table 5:** Stepwise linear regression analysis of factors associated with SARS-CoV-2 IgG titers

	B	Standard error	95% confidence interval for B		Beta	*P* value**
Vaccination type*	0.091	0.011	0.071	0.172	0.293	< 0.001
Charlson cormorbidity index^†^	− 0.052	0.021	− 0.094	− 0.011	− 0.083	0.014

Variables include sex, body mass index, vaccination type and Charlson Cormorbidity Index

*AZ–AZ based vaccine (reference) versus Medigen–Medigen based vaccines, BNT–BNT based vaccines, Moderna–Moderna-based vaccine. ^†^Charlson Cormorbidity Index: 0–1 (reference) versus 2–3 versus ≧ 4. Moderna: mRNA-1273 [Moderna]; AZ: ChAdOx1-S (AZD1222). **P for trend

## Discussion

In the current study, we demonstrated that the anti-spike protein antibody levels varied in Taiwanese subjects who received 3 doses of COVID-19 vaccination. Apart from the influence of different vaccines, patient characteristics such as age and gender may also determine vaccine responsiveness. Imperatively, subjects with concurrent morbidities had lower IgG levels. There was a dose-dependent effect on the CCI score and the antibody titer regardless of the vaccine type. The more comorbidities that Taiwanese subjects possessed, the poorer the vaccine responsiveness.

COVID-19 has caused colossal health and economic burdens worldwide since 2019. The first case of COVID-19 in Taiwan was diagnosed in January 2020. Taiwan had few domestic cases until small outbreaks occurred in May 2021 [7]. The dominant viral strains were the Alpha and Beta variants [3] before 2021, and the Delta variant cluster emerged in June 2021 [4] and was replaced by the Omicron variant in 2022 [8]. The strategy against the pandemic has changed from COVID-Zero to coexistence since March 2022 [16], after the broad coverage of the vaccination program in Taiwan. Herd immunity and vaccination are the two key strategies for fighting against the virus depending on vaccine availability or governmental policy. Nevertheless, immunity may be attenuated after vaccination. The vaccine booster strategy shall be crucial, particularly among immunocompromised subjects, such as liver transplantation recipients [10, 11], people living with human immunodeficiency virus [13] and uremic patients [12].

A third dose of COVID-19 vaccination has been adopted since 2021 around the world, which started in Israel [17]. The coverage rate of the 3rd dose of vaccination has been > 70% in Taiwan upon manuscript drafting. As denoted in the current study, mRNA vaccines are the most immunogenic and exert a higher serum IgG level [18]. The mix and match approach of SARS-CoV-2 vaccination yielded a stronger immune response, and Moderna-based vaccination seemed to yield higher antibody levels than AZ-based vaccination in Taiwanese people, as reported in previous studies [19, 20].

The clinical manifestations of COVID-19 infection vary from asymptomatic, flu-like symptoms, acute respiratory distress syndrome (ARDS), and pneumonia to death. The different clinical outcomes highlight that variable host immune responses against SARS-CoV-2 exist. In general, patients with underlying comorbidities, such as obesity, diabetes, decompensated liver disease, and immunocompromised status, are more likely to encounter severe COVID-19 or mortality than the general population [21, 22]. Kim et al. disclosed that concurrent comorbidities would determine severe COVID-19, and that the CCI score was the most critical predictor for mortality in a Korean cohort [23]. This raises the issue of whether different host immune responses exist after vaccination among subjects with variable patient characteristics or concurrent comorbidities. As with other studies, we found that age and male sex were two anthropological factors for the hypo-responsiveness to COVID-19 vaccination [24–26]. Another study interestingly showed that married and divorced males, but not singles and cohabitants, had a significantly lower SARS-CoV-2 IgG titer than females [27]. It has been postulated that estrogen, interacting with ESR1/2 receptors may potentially inhibit SARS-CoV-2 related immune response signaling in host cells. Besides the difference of socioeconomic status, whether estrogen is associated with different vaccine responses from the biological viewpoint remains unclear [28]. As this cohort could not represent the whole Taiwanese subjects, further large-scaled studies are warranted to clarify this point.

Whether subjects with more comorbidities have poor vaccine responses is elusive. In a Denmark study comprising mostly subjects with no (CCI = 0) or few comorbidities (CCI score = 1–2), those with a higher CCI score (> 2) had a poor spike IgG or spike-ACE2-receptor-blocking antibody response 3 months after one dose of vaccination [24]. Butt et al. also showed a lower vaccine effectiveness in subjects with a CCI score > 2 compared to those with a CCI score < 2 after 2 doses of vaccination [25]. Notably, the impact of concurrent comorbidities on vaccination response after 3 doses of COVID-19 vaccination has rarely been addressed. In the current study, we demonstrated that CCI scores were negatively correlated with anti-spike protein antibody levels regardless of vaccination type. With the emergence of viral variants, the current study opens the door for exploring the same issue regarding future vaccine inoculation strategies.

The current study has some limitations. We focused on timely vaccine response 1–3 months after 3 doses of vaccination, and the observation of the durability after a longer follow-up period is lacking. Despite we tried to exclude potential infected patients by selecting patients before the COVID-19 pandemic, a small proportion of the study cohort was not tested for nucleocapsid antibody. Due to the different vaccination strategies and combinations, the comparison with other ethnicities was also not feasible. In conclusion, subjects with more comorbidities were less responsive to 3-doses of COVID-19 vaccination serologically. With the innovation of next-generation vaccines and the emerging viral variants, studies regarding this issue in different populations and ethnicities are warranted in the future.

## Data Availability

The data and materials are available in Kaohsiung Medical University Hospital and could be obtained only with the approval of the corresponding author.

## Abbreviations

BNT: BNT162b2 [Pfizer-BioNTech]
AZ: ChAdOx1-S AZD1222
COVID-19: Coronavirus disease 2019
CCI: Charlson comorbidity index
SARS-CoV-2: Severe acute respiratory syndrome coronavirus 2
Moderna: MRNA-1273 [Moderna]
